# Role of adipose tissue GLP-1R expression in metabolic improvement after bariatric surgery in patients with type 2 diabetes

**DOI:** 10.1038/s41598-019-42770-1

**Published:** 2019-04-18

**Authors:** Miriam Ejarque, Fernando Guerrero-Pérez, Nuria de la Morena, Anna Casajoana, Nuria Virgili, Rafael López-Urdiales, Elsa Maymó-Masip, Jordi Pujol Gebelli, Amador Garcia Ruiz de Gordejuela, Manuel Perez-Maraver, Silvia Pellitero, Sonia Fernández-Veledo, Joan Vendrell, Nuria Vilarrasa

**Affiliations:** 1 Hospital Universitari de Tarragona Joan XXIII, Institut d’Investigació Sanitària Pere Virgili, Universitat Rovira i Virgili, Tarragona, Spain; 2 CIBER de Diabetes y Enfermedades Metabólicas Asociadas (CIBERDEM), Instituto de Salud Carlos III, Madrid, Spain; 3 Department of Endocrinology and Nutrition, Bellvitge University Hospital-IDIBELL, L’Hospitalet de Llobregat, Barcelona, Spain; 4 Bariatric Surgery Unit, Bellvitge University Hospital-IDIBELL, L’Hospitalet de Llobregat, Barcelona, Spain; 5 Department of Endocrinology and Nutrition and Health Sciences Research Institute and University Hospital Germans Trias i Pujol, Badalona, Spain

**Keywords:** Obesity, Type 2 diabetes

## Abstract

We aimed to explore the relationship between GLP-1 receptor (*GLP-1R*) expression in adipose tissue (AT) and incretin secretion, glucose homeostasis and weight loss, in patients with morbid obesity and type 2 diabetes undergoing bariatric surgery. RNA was extracted from subcutaneous (SAT) and visceral (VAT) AT biopsies from 40 patients randomized to metabolic gastric bypass, sleeve gastrectomy or greater curvature plication. Biochemical parameters, fasting plasma insulin, glucagon and area under the curve (AUC) of GLP-1 following a standard meal test were determined before and 1 year after bariatric surgery. *GLP-1R* expression was higher in VAT than in SAT. *GLP-1R* expression in VAT correlated with weight (r = −0.453, p = 0.008), waist circumference (r = −0.494, p = 0.004), plasma insulin (r = −0.466, p = 0.007), and systolic blood pressure (BP) (r = −0.410, p = 0.018). At 1 year, *GLP-1R* expression in VAT was negatively associated with diastolic BP (r = −0.361, p = 0.039) and, following metabolic gastric bypass, with the increase of GLP-1 AUC, (R^2^ = 0.46, p = 0.038). Finally, *GLP-1R* in AT was similar independently of diabetes outcomes and was not associated with weight loss after surgery. Thus, *GLP-1R* expression in AT is of limited value to predict incretin response and does not play a role in metabolic outcomes after bariatric surgery.

## Introduction

Glucagon-like peptide-1 (GLP-1) is a potent stimulator of glucose-dependent insulin secretion, and also confers beneficial actions on gastric emptying and appetite regulation^1–3^. GLP-1 exerts its effects principally through its interaction with the G protein-coupled receptor GLP-1R, which in humans is broadly expressed in the central nervous system and in peripheral tissues such as pancreas, heart, kidney, gastrointestinal tract and adipose tissue (AT)^4^. However, the extrapancreatic mechanisms and actions of GLP-1, especially in AT, remain insufficiently understood^5–9^. Accumulated data from *in vitro* studies in rat and human cell lines have described both lipogenic and lipolytic activity for GLP-1 that seems to be dependent on its concentration^5–8,10–12^. Moreover, studies in murine 3T3-L1 preadipocytes indicated that GLP-1 promotes pre-adipocyte differentiation and inhibition of apoptosis^13–15^. Interestingly, GLP-1R has been recently implicated in energy metabolism by directly stimulating mitochondrial bioenergetics and brown AT remodeling^16,17^. Some authors have hypothesized that promotion of adipogenesis through activation of GLP-1R increases the capacity of visceral hypertrophic adipocytes to store lipids, thereby decreasing ectopic lipid accumulation and improving insulin resistance (IR)^18^. In humans, GLP-1R expression was increased in visceral AT (VAT) from morbid obese subjects with a high insulin-resistant profile as compared with insulin-sensitive lean, overweight, and low insulin-resistant obese groups^8^. Furthermore, high GLP-1R expression in VAT predicted a greater improvement in the systemic IR index after biliopancreatic diversion surgery^8^.

In view of the aforementioned observations and considering that VAT dysfunction has been classically linked to inflammation and IR^19–21^, then it does not seem unreasonable to predict an additional role for this incretin receptor in AT metabolic functions, which might influence the recovery of glucose homeostasis and IR after massive weight loss. Accordingly, the aim of the present study was to explore the relationship between *GLP-1R* expression in AT and incretin secretion, glucose homeostasis, metabolism and weight loss after bariatric surgery. To do this, we used a well-characterized cohort of patients with morbid obesity and type 2 diabetes mellitus (T2D) undergoing bariatric surgery in the setting of a randomized controlled trial (RCT).

## Matherial and Methods

### Study design and setting

This study was part of a prospective single centre, non-blinded RCT, including patients with morbid obesity and T2D undergoing bariatric surgery. The trial was registered on January 25, 2016 at www.controlled-trials.com (ISRCTN14104758). Patients were consecutively recruited from the diabetes outpatient clinic of Bellvitge University Hospital, Barcelona, Spain. The study was conducted according to the principles of the Declaration of Helsinki. All patients signed an informed consent and the protocol study (PI11/01960) was approved by the Clinical Research Ethics Committee of Bellvitge University Hospital (reference PR 143/11).

The inclusion criteria were age between 18 and 60 years, body mass index (BMI) 35–43 kg/m^2^, T2D on hypoglycemic agents alone, insulin, or both. The exclusion criteria were the following: type 1 diabetes or positivity for glutamic acid decarboxylase autoantibodies, secondary forms of diabetes, acute metabolic complications, liver disease, renal dysfunction or patients under anticoagulant treatment, previous bariatric surgery, congenital or acquired abnormalities of the digestive tract, pregnancy, nursing or desired pregnancy in the 12 months following inclusion, and corticoid use by oral or intravenous route for more than 14 consecutive days in the last three months.

### Randomization

Patients were randomly assigned 1:1:1 to undergo metabolic Roux-en-Y gastric bypass (mRYGB), sleeve gastrectomy (SG) or greater curvature plication (GCP). The randomization process was performed by the statistics department at Bellvitge University Hospital. A computer random number generator with opaque sealed sequentially numbered envelopes with stratification according to baseline levels of HbA_1c_ (greater or lower/ equal to 7%) was used.

### Study protocol

In the mRYGB procedure, a 100-mL gastric pouch is created along with a 200-cm biliopancreatic limb and an alimentary limb of 100 cm. SG is a restrictive technique consisting of a gastric volume reduction of 75–80% by resecting the stomach over a 36-French catheter beginning 4 cm from the pylorus and ending at the angle of His. GCP is a reversible restrictive technique with no stomach resection, in which an invagination of the greater gastric curvature is performed with two running non-absorbable sutures, calibrated over a 36-French catheter. A physical examination with determination of anthropometrical parameters and a complete biochemical analysis was performed before bariatric surgery and at months 1, 3, 6, and 12 following the procedure.

Buse’s criteria were used to define T2D remission in each procedure at 1 year of follow-up: Complete remission = HbA_1c_ < 6% and fasting glucose < 100 mg/dl (<5.6 mmol/l) with no diabetes drugs for 1 year; partial remission = HbA1c < 6.5% and fasting glucose < 100–125 mg/dl (5.6–6.9 mmol/l) with no diabetes drugs for 1 year^22^.

### Anthropometric measurements

Body weight, height and waist circumference, and blood pressure were measured at every visit. Weight change after surgery was reported as total weight loss percentage (TWL%) [(preoperative weight − current weight)/(preoperative weight) × 100]. Body composition was analysed by dual-energy X-ray absorptiometry (Hologic QDR 4500; Hologic Inc., Waltham, MA) before and 12 months after surgery.

### Standard meal test

The standard meal test (SMT) was performed before bariatric surgery and at months 1 and 12. The SMT consisted of 200 ml of a liquid meal (Edanec®) containing 54% carbohydrates, 16% proteins, and 30% lipids, equivalent to 1 kcal/ml. Incretin-related treatments were discontinued at least 1 month before the analysis; the remainder of diabetes treatments, excluding insulin, were withdrawn at least 1 week before the SMT. Blood was drawn immediately before and 15, 30, 60 and 120 minutes following the SMT for determination of GLP-1 concentration^23^. Fasting glucagon levels were determined before the SMT. There were no changes to methods after trial commencement.

### Laboratory determinations

Glucose, cholesterol and triglycerides were determined using standard enzymatic methods. Plasma insulin was analysed by immunoassay (Coat-A-Count Insulin; Diagnostic Products Corp., Los Angeles, CA); inter- and intra-assay variability was 6.6–12.4% and 5.8–21.7%, respectively. GLP-1 was measured by radioimmunoassay (Millipore, Saint Charles, MO); inter and intra-assay variability was 10–23% and 22–38%, respectively. To obtain complete dipeptidyl peptidase IV inhibition, all samples were processed with a DPP-IV inhibitor (DPP4–010; Merck, Madrid, Spain). Glucagon was measured by enzyme immunoassay (Yanahaira Institute Inc., Awakura, Fujinomiya-shi Shizuoka, Japan).

### Gene expression analysis

Total RNA was extracted from subcutaneous AT (SAT) and VAT biopsies obtained during surgery using the RNeasy Lipid Tissue Mini Kit (Qiagen, Valencia, CA). One microgram of RNA was reverse transcribed with random primers using the reverse transcription system high capacity cDNA kit (Applied Biosystems, Foster City, CA). Quantitative *GLP-1R* expression was evaluated with Taqman low-density arrays (Hs00157705_m1; Applied Biosystems microfluidic cards) on a 7900HT Fast Real-Time PCR platform. The relative expression was measured using the 2^−ddCt^ method. Peptidyl-prolylisomerase A (*PPIA*, Hs04194521_s1) was used for normalization of gene expression levels and calculation of dCt values due to its stability across tissues and samples^24,25^.

### Outcomes

The primary outcome of the study was the predictive value of gut hormone dynamics (GLP-1, glucagon, peptide-YY [PYY] and ghrelin) on glucose metabolism improvement at 1 and 12 months after surgery for each procedure. The study had two main secondary outcomes. The first was to compare the rate of T2D remission in each procedure at 1 year of follow-up, defined by Buse’s criteria^22^. The results of the previous primary and secondary outcomes have been published^26^. The other secondary outcome was the determination of the role of GLP-1 R expression in AT in incretin response, weight loss, insulin sentivity improvement and diabetes remission after surgery. There were no changes to trial outcomes after commencement of the study.

### Statistical analysis

Based on preliminary data, the study was designed to detect a 20% difference in GLP-1 secretion (measured by the area under the curve [AUC] after the SMT) before and 1 month after bariatric surgery, with a power of 80% and α risk of 0.05. At least 10 subjects per group were considered, but to account for the possibility of loss at follow-up 15 patients per group were finally assigned. Biochemical and hormonal parameters were compared between procedures by analysis of variance (ANOVA). Categorical variables were compared using the Chi-square test and quantitative variables using ANOVA or the Kruskal-Wallis test. *GLP-1R* mRNA expression data were log transformed for analysis. GLP-1 AUC was calculated by the trapezoidal method^27^. Bivariate (Pearson or Spearman) and multivariate regression analyses were employed to determine the associations of *GLP-1R* expression in SAT and VAT with glucose, lipid profile, IR and incretin concentration. Moreover, the association of *GLP-1R* expression with T2D remission, homeostasis model assessment (HOMA)-IR and HOMA-β, weight loss and incretin secretion at 1 year was also tested. As this was an exploratory study, we did not make adjustments for multiple comparisons as the use of more stringent criteria could increase the likelihood of missing clinically significant associations between variables. Statistical analysis was performed using R software version 3.4.0 (R Foundation for Statistical Computing, Vienna, Austria). A *P*-value < 0.05 was considered statistically significant.

## Results

From May 2012 to February 2014, 45 morbidly obese patients with T2D, aged 49.4 ± 7.9 years, BMI 39.4 ± 1.9 kg/m^2^, initial HbA_1c_ 7.7 ± 1.9%, were consecutively randomized to mRYGB, SG or GCP (n = 15 in each group). All patients completed the study with the exception of one patient allocated to the SG group who withdrew from the study after 10 months. Paired samples from SAT and VAT were obtained during surgery in 40 patients (n = 15 mRYGB, n = 13 SG and n = 12 GCP), and all patients completed the 12 months study period. Baseline clinical and biochemical characteristics of the 40 patients are listed in Table 1.

**Table 1:** Baseline characteristics of patients.

Parameter	Metabolic Gastric Bypass	Sleeve Gastrectomy	Greater Curvature Plication	p*-*value
Number of patients	15	13	12	
Sex (male/female)	7/8	4/9	2/10	0.301
Age, yr	51.10 (7.70)	48.20 (9.47)	49.60 (8.94)	0.692
Weight, kg	103 (10.8)	103 (11.2)	105 (13.2)	0.883
Body mass index, kg/m^2^	38.7 (2.01)	39.3 (1.42)	40.4 (1.35)	0.037*
Waist circumference, cm	118 (7.58)	118 (7.99)	117 (8.48)	0.878
Total body fat, %	38.4 [28; 43]	35.8 [31; 37]	37.8 [36; 41]	0.301
T2D duration, months	54 [39.5–114]	120 [51.2–180]	83 [45–144]	0.216
C-peptide, ng/ml	3.04 ± 1.01	3.13 ± 1.80	3.75 ± 2.75	0.566
Fasting glucose, mmol/l	8.38 (3,00)	1.08 (0.64)	1.26 (1.00)	0.639
HbA_1c_, %	7.39 (1.95)	7.94 (1.79)	8.47 (2.19)	0.377
Insulin treatment, %, (n)	33.30 (5)	38.5 (5)	58.3 (7)	0.400
Dyslipidemia, %, n	73.3 (11)	84.6 (11)	91.7 (11)	0.518
Hypertension, %, n	66.7 (10)	69.2 (9)	75.0 (9)	0.912

Data are expressed as mean ± SD for normally distributed variables and median [interquartile range] for non-normally distributed variables. *p < 0.05 was considered statistically significant. T2D: type 2 diabetes; HbA_1c_: glycated hemoglobin A1c.

As a summary of previously published data^26^, TWL% was significantly greater in the mRYGB group than in SG and GCP groups at 12 months: −35.29 ± 8.17 vs. −27.26 ± 5.66 vs. *−*20.24 ± 7.49%, respectively, p < 0.05. At 1 year of follow-up, HbA_1c_ was significantly lower in the mRYGB group than in the SG and GCP groups: 5.09 (0.62) vs. 6.24 (0.85) vs. 6.79 (1.39) %, respectively, p < 0.05. After surgery, the increase in the AUC for GLP-1was significantly higher in the mRYGB group than in SG and GCP groups: 87.3 [6.21;163] vs. 22.2 [2.40;32.9] vs. −0.28 [−23.94;40.5], respectively, p < 0.05. Short-term complete diabetes remission at 1 year was found in 80% of patients in the mRYGB group (n = 12), 53.8% in the SG group (n = 7) and 16.7% in the GCP group (n = 2), p < 0.001.

**Figure 1:**
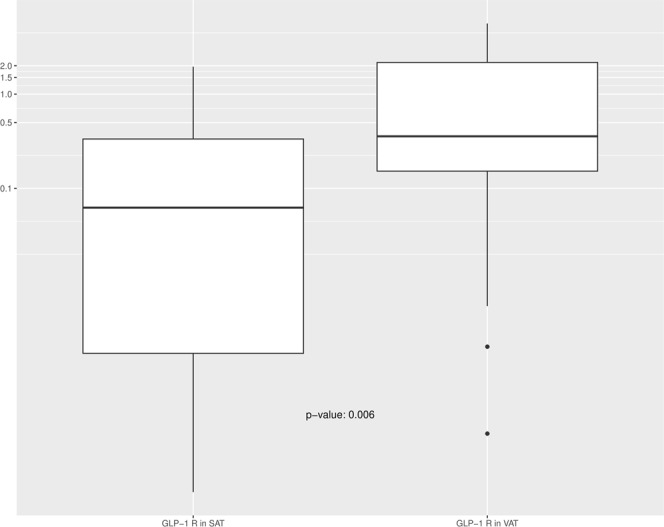
Comparison of *GLP-1R* expression in visceral (VAT) and subcutaneous (SAT) adipose tissue.

**Figure 2:**
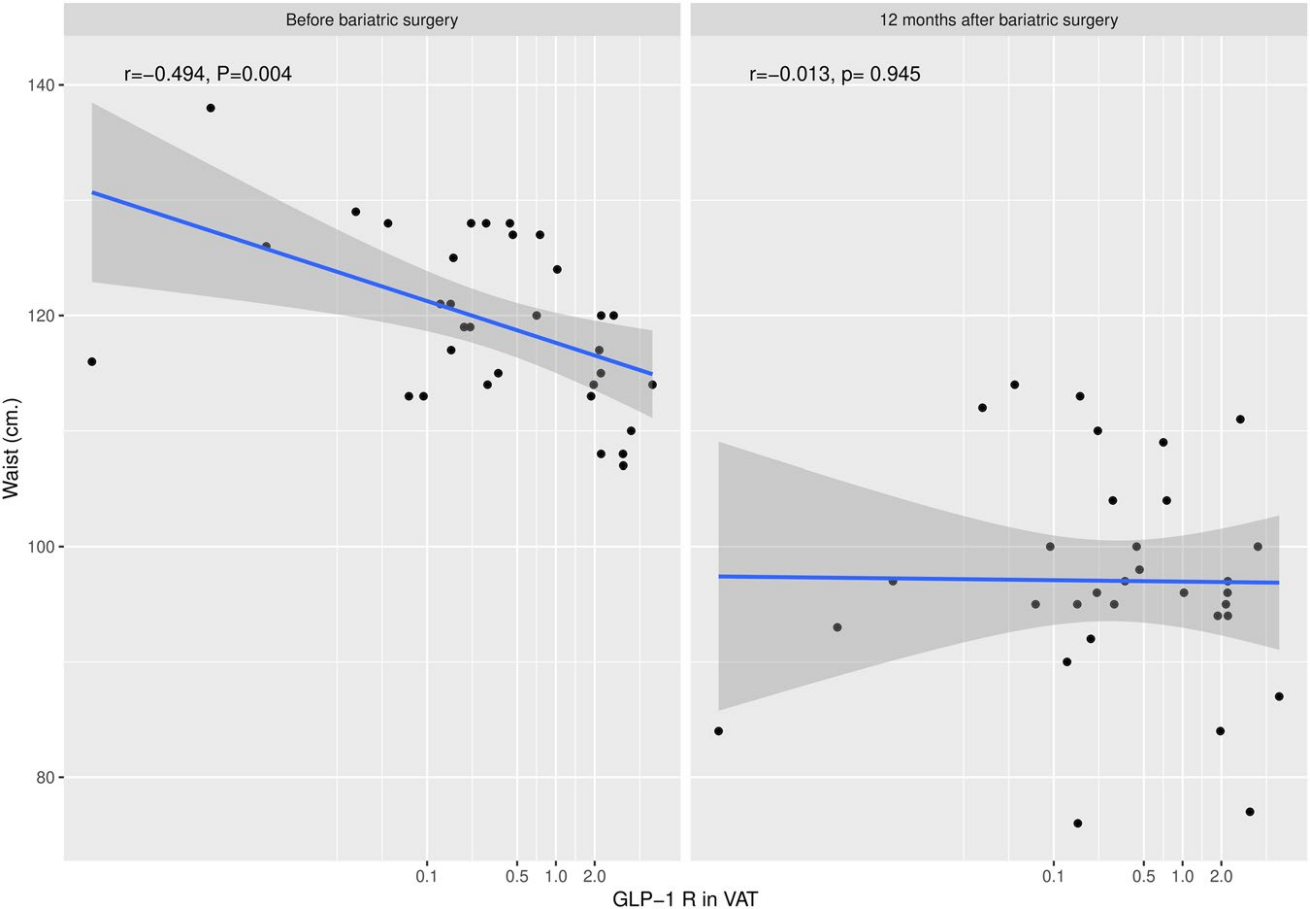
Correlative analysis of *GLP-1R* mRNA expression in visceral (VAT) with waist circumference before and 12 months after surgery. The correlations were determined by Pearson’s correlation coefficient test (r).

Analysis of *GLP-1R* expression in AT biopsies at surgery revealed a higher expression of the receptor in the visceral than in the subcutaneous depots (Fig. 1). Nonetheless, when we analysed *GLP-1R* expression in relation to patients’ baseline characteristics, VAT *GLP-1R* expression was inversely correlated with weight (r = −0.453, p = 0.008) and waist circumference (r = −0.494, p = 0.004), indicating that patients with greater weight and waist circumference had lower *GLP-1R* expression in this depot (Fig. 2). No association was found between *GLP-1R* expression in VAT and other anthropometrical parameters such as body fat or BMI. Regarding biochemical parameters, VAT *GLP-1R* expression was inversely correlated with basal plasma insulin (r = −0.466, p = 0.007), indicating that patients with higher insulin concentrations had lower *GLP-1R* expression. No differences in VAT *GLP-1R* expression were found with glucose metabolism parameters (glucose and HbA_1c_), HOMA-IR, HOMA-β or lipid profile. Similarly, there was no correlation between VAT *GLP-1R* expression and pre-surgical GLP-1 AUC in plasma or fasting glucagon concentrations. Regarding blood pressure, VAT *GLP-1R* expression was inversely correlated with systolic blood pressure (r = −0.410, p = 0.018) (Table 2). A multivariate regression analysis to account for confounders was performed finding that VAT *GLP-1R* expression remained inversely correlated with weight (R^2^ = 0.223, p = 0.049) after adjusting for gender, BMI, insulin and HbA_1c_. Analysis of *GLP-1R* expression in SAT showed no significant associations with anthropometrical, biochemical or hormonal parameters.

**Table 2:** Bivariate correlations between *GLP-1R* expression in visceral (VAT) and subcutaneous (SAT) adipose tissue and baseline plasma metabolic and hormonal parameters.

	*GLP-1 R* in VAT		*GLP-1 R* in SAT	
	R	p-value	R	p-value
**Weight**	−0.453	0.008*	−0.121	0.495
**Glucose**	0.069	0.703	−0.385	0.240
**HOMA-IR**	−0.198	0.276	−0.210	0.241
**HbA**_**1c**_	−0.169	0.347	−0.132	0.457
**C-peptide**	0.148	0.412	−0.078	0.660
**Glucagon**	−0.101	0.575	−0.109	0.539
**GLP-1 AUC**	0.300	0.108	−0.034	0.860
**Total cholesterol**	0.036	0.841	−0.094	0.598
**HDL cholesterol**	0.047	0.796	−0.054	0.761
**LDL cholesterol**	−0.188	0.347	−0.009	0.963
**Triglycerides**	0.197	0.272	0.063	0.725
**sBP**	−0.410	0.018*	0.094	0.597
**dBP**	−0.313	0.076	0.041	0.817

HOMA-IR: homeostasis model assessment insulin resistance; GLP-1 AUC (area under the curve for GLP-1); HbA_1c_: glycated hemoglobin A1c; sBP: systolic blood pressure; dBP: diastolic blood pressure; HDL: high-density lipoprotein; LDL: low-density lipoprotein. *p < 0.05 was considered statistically significant.

When we analysed AT *GLP-1R* expression using parameters at 12 months after surgery, we found an inverse association between *GLP-1R* expression in VAT and diastolic blood pressure at 12 months (r = −0.361, p = 0.039), however this correlation lost significance in the multivariate regression analysis after adjusting for gender, BMI, HbA_1c_ and type of surgery. No other correlations were found between VAT or SAT *GLP-1R* expression in the bivariate and multiple regression analysis and glucose metabolism parameters or lipid profile at 12 months (Table 3), or with their changes after the intervention.

**Table 3:** Bivariate correlations between *GLP-1R* expression in visceral (VAT) and subcutaneous (SAT) adipose tissue and plasma metabolic and hormonal parameters measured 12 months after bariatric surgery.

	*GLP-1 R* in VAT		*GGLP-1 R* in SAT	
	R	p-value	R	p-value
**Weight**	−0.205	0.253	0.047	0.793
**Glucose**	−0.110	0.541	−0.067	0.705
**HOMA-IR**	−0.023	0.899	−0.295	0.096
**Insulin**	−0.067	0.717	−0.032	0.858
**HbA**_**1c**_	0.062	0.734	−0.179	0.311
**GLP-1 AUC**	−0.119	0.524	0.117	0.516
**Total cholesterol**	0.140	0.438	0.008	0.963
**HDL cholesterol**	0.170	0.345	−0.027	0.879
**LDL cholesterol**	−0.079	0.680	−0.014	0.939
**Triglycerides**	0.134	0.457	0.045	0.799
**sBP**	−0.219	0.221	0.054	0.762
**dBP**	−0.361	0.039*	0.199	0.259

HOMA-IR: homeostasis model assessment insulin resistance; GLP-1 AUC (area under the curve for GLP-1); HbA_1c_: glycated hemoglobin A1c; sBP: systolic blood pressure; dBP: diastolic blood pressure; HDL: high-density lipoprotein; LDL: low-density lipoprotein. *p < 0.05 was considered statistically significant.

**Figure 3:**
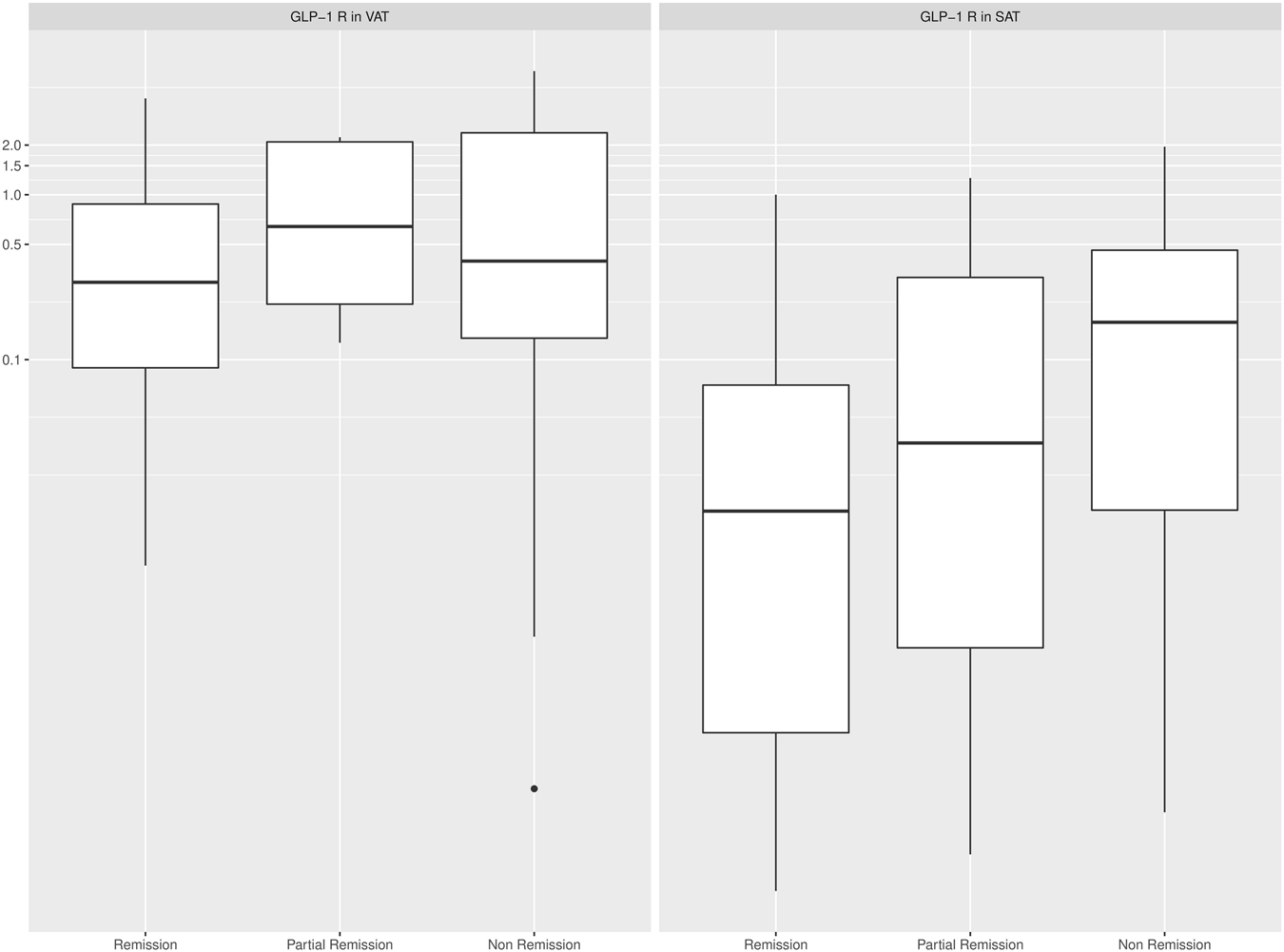
Comparison of *GLP-1R* expression in visceral (VAT) and subcutaneous (SAT) adipose tissue and diabetes outcomes 12 months after surgery.

Regarding the association between *GLP-1R* expression in AT and GLP-1 concentration, only those patients undergoing mRYGB, characterized by a greater metabolic improvement and higher incretin response, showed a negative relationship between *GLP-1R* expression in VAT and the increase of GLP-1 AUC at 1 year after surgery (r = −0.670, p = 0.015). In multiple regression analysis, VAT *GLP-1R* expression after mRYGB remained inversely associated with the increase of GLP-1 AUC at 1 year after surgery, after adjusting for changes in weight, BMI and HbA_1c_ (R^2^ for the model 0.46, p = 0.038). However, *GLP-1R* expression in AT did not predict metabolic outcomes. Accordingly, no correlations were observed between *GLP-1R* expression in AT and glucose, HbA_1c_ or insulin sensitivity improvement calculated by HOMA-IR or HOMA-β after the intervention. Moreover, *GLP-1R* expression at VAT and SAT did not differ between patients remaining with T2D compared with those under partial or complete T2D remission after the bariatric procedure: 0.29 (0.04;0.82) vs. 1.05 (0.22;2.10) vs. 0.28 (0.08;2.18), p = 0.779 and 0.02 (0.0009;0.007) vs. 0.03 (0.0026;0.32) vs. 0.17 (0.01;0.37), p = 0.278, respectively (Fig. 3). Finally, *GLP-1R* expression in AT was not associated with weight response after surgery.

## Discussion

In the present study, we evaluated the clinical significance of *GLP-1R* expression in AT in relation to plasma incretin concentration, glucose homeostasis, insulin sensitivity improvement and weight outcome in patients with T2D undergoing bariatric surgery in an RCT. In subjects randomized to mRYGB, basal *GLP-1R* expression in VAT was an inverse determinant of the increase in incretin concentration after surgery. However, *GLP-1R* expression in AT was not associated with diabetes remission or weight loss after surgery.

GLP-1R is broadly expressed in many tissues beyond the pancreas, including lung, brain, stomach, heart, kidney, endothelium, bone and also AT^4^. The physiological actions of GLP-1R have been well documented over the last decade, especially after the extensive clinical applications of GLP-1 mimetics for diabetes, such as GLP-1R agonists and DPP-4 inhibitors^1,2^. However, the significance of GLP-1R in AT is not fully understood and remains contentious. Data from *in vitro* studies indicate that GLP-1 has a role in adipogenesis^5–15^. Murine 3T3-L1 adipocyte precursors treated with GLP-1 and exendin-4 (a GLP-1R agonist) showed increased differentiation^14^, favouring intracellular lipid accumulation and insulin-mediated glucose uptake through up-regulation of Glut-4 expression and phosphorylation of the insulin receptor in adipocytes^28^. While the evidence is less established in humans, in mature adipocytes GLP-1 treatment induced a significant lipolytic effect in a dose-dependent manner^8,12^. More recently, treatments with GLP-1 analogues have been associated with increased proliferation and inhibition of apoptosis in human adipose-derived stromal cells^29^, and also with stimulation of mitochondrial bioenergetics in AT^16,17^.

In addition to mature adipocytes, AT also contain various cell populations in the stromal vascular fraction (SVF), including mesenchymal stem cells (MSCs), preadipocytes, fibroblasts, vascular endothelial cells, and a range of immune cells such as AT macrophages. GLP-1R has been found expressed in mature adipocytes and MSCs, but the expression in the rest of SVF components remain elusive^8^. The GLP-1R agonist exendin-4 is able to inhibit LPS-induced TNF-α expression in macrophages, which is an indirect evidence of the anti-inflammatory role of GLP-1 in macrophages^30^. Also, inhibition of nuclear factor-kappa B (NF-κB) activation of macrophage population in AT in the presence of GLP-1R analogs has been reported^31^. Furthermore, endothelial vascular cells are sensitive to the GLP1-R analogs, with a net anti-inflammatory effect through different molecular mechanisms driven by the activation of adenosine monophosphate-activated protein kinase (AMPK)^32^. Altogether, previous data would suggest that GLP-1R expression within AT is due to the contribution of its two major components, mature adipocytes and the cellular components of the SVF, with a predominant anti-inflammatory effect in the last location.

GLP-1R expression has been described in AT of lean subjects, with higher expression in visceral than in subcutaneous depots, and with lower expression than in insulin-resistant morbidly obese subjects^8^. In line with a previous report, we observed higher expression of *GLP-1R* in VAT than in SAT compartments^8^. Consistent with this, a recent study identified *GLP-1R* expression in epicardial AT, which represents the visceral fat equivalent of the heart^33^. Interestingly, an excess of epicardial fat has been implicated in IR and cardiovascular diseases, exerting its atherogenic action through the paracrine secretion of pro-inflammatory cytokines^34^. Similarly, an increase in abdominal VAT is associated with an increased metabolic, cardiovascular risk and greater mortality prediction capacity^20,21,35^. Many of these deleterious events are mediated, in part, by inflammatory mechanisms originating in the AT depots. Indeed, recent data suggest that GLP-1-based therapies may have anti-inflammatory effects in several organs including AT, by reducing the production of pro-inflammatory cytokines and infiltration of immune cells^31,36^. Therefore, improving the knowledge of the regulatory dynamics of the GLP1/GLP-1R axis in AT might have relevance for the treatment of local and systemic inflammatory events in obesity and T2D.

In patients with morbid obesity without T2D, the expression of *GLP-1R* in VAT has been described to be positively associated with the degree of IR and with the greatest insulin sensitivity improvement after biliopancreatic diversion surgery^8^. While our results in patients with T2D support this association, our findings revealed a negative correlation between insulin levels and *GLP-1R* expression in VAT. This is consistent with the negative relationship observed between weight, waist circumference and blood pressure and *GLP-1R* expression in this depot; thus, the greater the metabolic disturbances the lower the *GLP-1R* expression in AT. It should be highlighted that our cohort comprised patients with morbid obesity and T2D, and this may result in a different behaviour when compared with euglycemic subjects. Indeed, to the best of our knowledge, this is the first study to analyse the GLP-1/GLP-1R axis in AT of subjects with these characteristics. Our results lead us to speculate that our patient cohort might present disrupted GLP-1/GLP-1R signalling, resulting in a decrease in GLP-1 sensitivity in AT. This hypothesis might explain why those patients with greater BMI and levels of insulin, who would be expected to have the lowest circulating GLP-1 concentrations and a higher expression of the receptor, showed the opposite; that is, a lower receptor expression at the VAT level.

Regarding the relationship between *GLP-1R* expression in AT and GLP-1 concentration/secretion, we were unable to find an association before surgery. However, in subjects who underwent mRYGB, who showed the greatest incretin response after surgery, *GLP-1R* expression in AT negatively predicted the increase in GLP-1 concentration after the procedure. Although this finding has to be interpreted with caution due to the small sample size, it might suggest the existence of a regulatory feedback between GLP-1 secretion and *GLP-1R* expression in VAT. The absence of an association in the other bariatric procedures could be due to the lower improvement in the incretin response observed in non-derivative surgeries.

GLP-1R has been associated with both lipogenic and lipolytic activity^5–15^. In the present study, however, no association was found between *GLP-1R* expression and plasma lipid profile before or after surgery. Nevertheless, we only determined plasma circulating lipids and not intra-adipocyte lipolysis.

Many hormonal and clinical factors have been implicated in glucose improvement and weight loss after bariatric surgery^37–39^. To our knowledge, however, this is the first study analysing the predictive value of pre-surgery *GLP-1R* expression in AT on glucose metabolism and weight outcomes after different bariatric procedures. But, despite its potential relationship with incretin secretion, no association was found between *GLP-1R* expression in AT and either diabetes remission, insulin resistance improvement or weight loss after surgery.

We are aware that the study has some limitations. The limited number of subjects included and the low *GLP-1R* expression levels detected may reduce the strength of our observations. Moreover, the fact that AT biopsy samples were only available during surgery, and not afterwards, prevents the evaluation of GLP-1R expression changes with metabolic improvement after bariatric surgery, thus limiting our results. Also, we could only analyse glucagon concentrations in the fasting state, and so we could not calculate the AUC for this hormone, which is a more reliable indicator of its secretion. As our study was exploratory, further additional dedicated studies should be conducted in larger cohorts with pre-planned hypotheses and higher statistical power to confirm the observed associations.

## Conclusions

In subjects with morbid obesity and T2D, *GLP-1R* expression in AT is of limited value to predict incretin response and does not play a role in metabolic outcomes such as insulin sensitivity improvement, T2D remission and weight loss after bariatric surgery. Further studies are warranted to explore a possible paracrine role of this receptor in AT.

## Data Availability

All data analysed during this study is included in this article as well as in a previous published article cited in the references. If any further information is required please contact the corresponding author.
